# Patient Characteristics Associated With Phone and Video Visits at a Tele-Urgent Care Center During the Initial COVID-19 Response: Cross-Sectional Study

**DOI:** 10.2196/50962

**Published:** 2024-01-19

**Authors:** Saif Khairat, Roshan John, Malvika Pillai, Philip McDaniel, Barbara Edson

**Affiliations:** 1 Carolina Health Informatics Program University of North Carolina at Chapel Hill Chapel Hill, NC United States; 2 Cecil G Sheps Center for Health Services Research Chapel Hill, NC United States; 3 School of Nursing University North Carolina at Chapel Hill Chapel Hill, NC United States; 4 Digital Research Services Department University of North Carolina at Chapel Hill Chapel Hill, NC United States; 5 UNC Health Morrisville, NC United States

**Keywords:** telehealth, telemedicine, tele-urgent care, virtual urgent care, nonemergency care, televisit, phone visit, video visit, urgent care, health services research, COVID-19, health disparities, insurance status, cross-sectional study

## Abstract

**Background:**

Health systems rapidly adopted telemedicine as an alternative health care delivery modality in response to the COVID-19 pandemic. Demographic factors, such as age and gender, may play a role in patients’ choice of a phone or video visit. However, it is unknown whether there are differences in utilization between phone and video visits.

**Objective:**

This study aimed to investigate patients’ characteristics, patient utilization, and service characteristics of a tele-urgent care clinic during the initial response to the pandemic.

**Methods:**

We conducted a cross-sectional study of urgent care patients using a statewide, on-demand telemedicine clinic with board-certified physicians during the initial phases of the pandemic. The study data were collected from March 3, 2020, through May 3, 2020.

**Results:**

Of 1803 telemedicine visits, 1278 (70.9%) patients were women, 730 (40.5%) were aged 18 to 34 years, and 1423 (78.9%) were uninsured. There were significant differences between telemedicine modalities and gender *(P<*.001), age *(P<*.001), insurance status *(P<*.001), prescriptions given *(P<*.001), and wait times *(P<*.001). Phone visits provided significantly more access to rural areas than video visits *(P<*.001).

**Conclusions:**

Our findings suggest that offering patients a combination of phone and video options provided additional flexibility for various patient subgroups, particularly patients living in rural regions with limited internet bandwidth. Differences in utilization were significant based on patient gender, age, and insurance status. We also found differences in prescription administration between phone and video visits that require additional investigation.

## Introduction

Health systems rapidly adopted telemedicine as an alternative health care delivery modality in response to the COVID-19 pandemic. Demographic factors, such as age and gender, may play a role in patients’ choice of a phone or video visit [1-3]. However, it is unknown whether there are utilization differences between phone and video visits.

The pandemic led to a rise in phone and video consultations, providing an opportunity to study their usage across demographics and outcomes, such as medication prescriptions. Telemedicine can help improve health access and reduce disparities for vulnerable populations [4-7]. Although we know that medication prescription differs between in-person and video visits [8], there is a gap in the knowledge regarding differences in prescription administration, whether medication was prescribed or not, between telephone and video visits. Driven by prior differences in prescription administration among providers based on gender and specialty [7], we hypothesized that prescription administration, a service outcome of telemedicine, may differ between phone and video visits.

Phone-based treatment has been found feasible, acceptable, and effective compared to face-to-face visits. It is a promising alternative in telemedicine, offering tailored interventions [9]. Phone visits have taken less time and have been used more frequently, but there have not been significant differences in patient perceptions or other clinical outcomes [10].

Telemedicine’s growth during the pandemic has led to a need for understanding the limitations of telephone-based versus video-based consultations for clinical care [11,12]. Patients reported that video consultations were more favorable compared to phone consultations, claiming that video visits led to improved outcomes, better diagnostic accuracy, and patient satisfaction [13-18].

Previous studies have looked at the impact of phone or video visits on vulnerable patients [19-23], but there is a lack of research on the differences in patient characteristics between the 2 modes of telemedicine-based care. Understanding these differences can help health organizations and policy makers tailor telehealth options to better suit patients.

Telemedicine use during the pandemic has been examined in various clinical environments, such as primary care, geriatrics, and subspecialties [5,22,23]. It is unclear how phone and video health care delivery in urgent care clinics was affected during the initial phases of the COVID-19 pandemic, especially regarding wait times and visit duration. The demand for urgent care clinics increased due to emergency department overcrowding, cost increase, and long wait times [24,25]. Therefore, it is important to understand the changes in urgent care practices considering telemedicine deployment postpandemic.

In this exploratory study, we examined patient and service characteristics of on-demand telehealth utilization and whether they differed by modality during the initial phase of the pandemic when the health care system suspended all in-clinic visits. We used the Donabedian framework of structure-process-outcome to inform this study design [26].

## Methods

### Study Overview

We conducted a descriptive analysis on a cross-sectional study of patients using a statewide, on-demand tele-urgent care clinic in the southeastern United States region. The Virtual Urgent Clinic (VUC) is an on-demand clinic open for nonemergency concerns 24 hours a day and 7 days a week. Regardless of whether they are new or existing patients, any individuals can register and access the virtual clinic through the web-based portal. To use the telemedicine service, individuals must create an account, input their medical history, and request a virtual care visit. Individuals can choose their telemedicine modality—telephone or video—through a computer, tablet, or phone.

The cost of the visit was the same for phone and video visits. The clinic provides on-demand service such that individuals can log on to the web-based portal and choose to have a visit immediately or schedule a visit for a later date. Board-certified physicians are available 24 hours every day of the week to provide care for patients. If an individual is an existing patient, documentation of the virtual visit is integrated into the electronic medical record after the visit is completed.

### Data Collection

VUC monthly data were collected from March 3, 2020, through May 3, 2020, using the institutional data warehouse. The data set included patient information, such as age, gender, insurance status, and residential address, and service characteristics, such as telemedicine modality, wait time, visit duration, and medication prescription outcomes. To avoid double counting of patients or visits, each patient and each visit received a unique identifier. Incomplete encounters were recorded in the data set as incomplete if the call was not completed for any reason. The rate of incomplete encounters was only 7.9% (142/1803) of the total visit volume in this study and was included to better understand the characteristics of patients who sought care via telehealth.

### Outcomes

Our primary endpoints were the characterization of telemedicine modalities (phone vs video) on patient characteristics measured by demographics and insurance status, utilization measured by the volume of visits; and service characteristics measured by medication prescriptions and visit wait times. The secondary endpoint was utilization, which was measured by the number of visits from rural and urban neighborhoods.

### Statistical Analysis

The study data included patient age, gender, health insurance status, address, number of medication prescriptions, number of visits, and choice of telemedicine modality. For each of these variables, we calculated descriptive statistics for each demographic category stratified by modality (phone or video) and the total of both groups. A *χ*^2^ test was calculated to check for significant differences between telemedicine visits and these variables. Additionally, we calculated the average wait time and visit duration for phone and video visits. A 2-sample *t* test assuming unequal variances (Welch *t* test) was also conducted to determine if there was a statistically significant difference in the average wait times and visit duration lengths between phone and video telemedicine visits.

To examine the predictors of prescription administration, we constructed a logistic regression model with a dichotomous dependent variable of prescription administration (0=no prescription=0 and 1=at least 1 prescription given) as a dependent outcome variable and patient age, gender, insurance status, location, and telemedicine modality as independent variables in the model predictors. We used a *P* value level of .05 to indicate statistical significance.

### Geospatial Analysis

Geographical locations for patients with VUC visits over the phone or video were examined to assess the urban-rural spread of the patients in this data set. Using the US Census definition, cities with populations of 50,000 people or more were designated as urban, and those with less than 50,000 people were designated as rural. In the telemedicine data set, 198 places in North Carolina were found, of which 179 were classified as rural and 19 were classified as urban, which was used to develop the health access map. A *χ*^2^ analysis was used to determine the significance between an encounter from an individual in an urban or rural area and the encounter modality.

To understand the association between telehealth modality and location, we used ArcGIS (Esri) to map zip code–level populations, as reported in the 2010 US Census Bureau data, with VUC visits based on Zip Code Tabulation Areas (ZCTAs). We used the 2016 American Community Service (ACS) to calculate the percentage of households with internet access by ZCTA. We then mapped the ACS data and visit counts from the VUC by modality on the North Carolina (NC) map to better understand the preference of patients for modalities based on internet availability.

We used natural breakdowns to quantify the percentage of households with internet in each NC zip code to determine the threshold for low, medium, and high categories based on the 2016 ACS data set. The colors along the bottom row (gray to light blue to teal) represent ZCTAs with a low percentage (0%-71%) of households with internet access and an increasing number of phone (or video) visits. The colors in the middle row (light pink to light purple to blue) represent ZCTAs with a medium percentage (72%-82%) of households with internet access and an increasing number of phone (or video) visits. The colors along the top row (pink to purple to dark purple) represent ZCTAs with a high percentage (83%-100%) of households with internet access and an increasing number of phone (or video) visits. The colors along the diagonal (gray to light purple to dark purple) represent ZCTAs with low internet access and low telemedicine visits, medium internet access and medium telemedicine visits, and high internet access and high telemedicine visits. For phone visits, the breaks were 1-2 (low), 3-6 (medium), and 7-37 (high). For video visits, the breaks were 1 (low), 2-3 (medium), and 4-15 (high). We used quantiles to determine the threshold for low, medium, and high categories based on the ACS 5-year estimates from 2015-2019.

### Ethical Considerations

University of North Carolina at Chapel Hill institutional review board approval was obtained prior to conducting this study (18-1628).

## Results

### Telemedicine Visit Overview

Table 1 shows a series of visit counts of the patients who used the telemedicine service during the observed period categorized by the patient characteristics captured in this study. It also indicates the *χ*^2^ and *P* values for significance tests for the differences between these observed characteristics.

**Table 1:** Percentage statistics and χ^2^ values for phone and video telemedicine visits.

Demographic			Phone visits (n=1414)		Video visits (n=389)		Total visits (N=1803)		Chi-square (df)			*P* value	
Visits per day, mean (SD)			22.8 (9)		6.3 (3.1)		29.1 (10.7)		N/A^a^			N/A	
**Gender, n (%)**										16.79 (2)			<.001
	Women	1033 (73)		245 (63)		1278 (70.9)							
	Men	377 (26.7)		144 (37)		521 (28.9)							
	Nonbinary	4 (0.3)		0 (0)		4 (0.2)							
**Age (years), n (%)**										24.99 (4)			<.001
	<18	96 (6.8)		57 (14.7)		153 (8.5)							
	18-34	579 (40.9)		151 (38.8)		730 (40.5)							
	35-50	486 (34.4)		123 (31.6)		609 (33.8)							
	51-64	185 (13.1)		44 (11.3)		229 (12.7)							
	≥65	68 (4.8)		14 (3.6)		82 (4.5)							
**Health insurance status, n (%)**										18.91 (1)			<.001
	Insured	329 (23.3)		51 (13.1)		380 (21.1)							
	Uninsured	1085 (76.7)		338 (86.9)		1423 (78.9)							
**Residence, n/N (%)**										6.74 (1)			.009
	Rural	782/1370 (57.1)		189/381 (49.6)		971/1751 (55.5)							
	Urban	588/1370 (42.9)		192 /381 (50.4)		780/1751 (44.5)							
**Prescriptions per visit, n (%)**										24.07 (1)			<.001
	Received	980 (69.3)		218 (56)		1198 (66.4)							
	Did not receive	434 (30.7)		171 (44)		605 (33.6)							

^a^N/A: not applicable.

### Patient Characteristics

Phone visits constituted most of the 1803 total visits (n=1414, 78.4%), with an average of 22.8 (SD 9) daily visits, while video visits accounted for the remaining visits (n=389), with a daily average of 6.3 (SD 3.1) visits. Most of the patients were women across both phone and video modalities (phone visits: n=1033, 73%; video visits: n=245, 63%). Among age groups, patients aged 18 to 34 years had the most visits (phone visits: n=579, 40.9%; video visits: n=151, 38.8%), with patients aged 35 to 50 years being the next most represented age group (phone visits: n=486, 34.4%; video visits: n=123, 31.6%). Across both modalities, the least present age group included patients older than 65 years (phone visits: n=68, 4.8%; video visits: n=14, 3.6%). Most patients across both modalities were uninsured (phone visits: n=1085, 76.7%; video visits: n=338, 86.9%).

Significant differences between telemedicine modalities and gender *(P<*.001), age (*P<*.001), insurance status (*P*<.001), health access (*P*=.009), and prescriptions given (*P*<.001). This suggests that men, patients younger than 18 years, uninsured patients, and patients residing in urban areas preferred the video modality for telemedicine visits, and video visits were more associated with not getting prescriptions.

### Telemedicine Service Characteristics

#### Prescription Administration

More patients received at least 1 prescription (phone visits: n=980, 69.3%; video visits: n=218, 56%) from a telemedicine visit rather than no prescription. Video visits were more associated with no prescriptions than phone visits (*P*<.001). Significant differences were found in medication prescription administration between phone and video visits (*P*<.001; Table 1).

For phone visits, of a total of 1414 phone visits, 980 (69.3%) resulted in at least 1 prescription given, while the other 434 did not receive any prescriptions. On average (SD), patients received 1 (1.02) prescription per encounter. Of all phone visits, 434 (30.7%) patients did not receive a prescription, 944 (66.8%) patients received 1-3 prescriptions in an encounter, and 36 (2.5%) patients received 4-7 prescriptions in an encounter.

For video visits, from a total of 389 video visits, 218 (56%) resulted in at least 1 prescription given, while the other 171 did not receive any. The average (SD) number of prescriptions per encounter was 0.84 (1.00). Of all video visits, 171 (43.9%) patients did not receive a prescription, 210 (54%) patients received 1-3 prescriptions in an encounter, and 8 (2.1%) patients received 4-7 prescriptions in an encounter.

We found that 5 patient characteristics were strong predictors of telemedicine prescription administration (Table 2). Predictors that were positively associated with prescription administration were patients aged 18 to 34 years (β=.62, *P*<.001), 35 to 50 years (β=.81, *P*<.001), and older than 65 years (β=.94, *P*=.002). Predictors that were negatively associated with prescription administrations were video visits (β=–.47, *P*<.001) and male patients (β=–.38, *P*<.001). There was no significant relationship between patients’ insurance status and prescription rates.

**Table 2:** Logistic regression model showing patient demographic associations with telemedicine prescription administration. The independent variables were modality, age, gender, and insurance status. The depended variable was prescriptions given.

			Estimate		SE		*z* score		Pr(>|z|)^a^		*R* ^2^	
Model intercept			0.2711		0.1853		1.463		.14		0.027269	
**Modality**												
	Video	-0.4724		0.1204		-3.922		<.001		N/A^b^		
**Gender**												
	Men	-0.3878		0.1108		-3.5		<.001		N/A		
	Nonbinary	0.4606		1.1715		0.393		.69		N/A		
**Health insurance status**												
	Insured	0.1629		0.1308		1.245		.21		N/A		
**Age (years)**												
	18-34	0.6227		0.1858		3.351		<.001		N/A		
	35-50	0.8057		0.19		4.241		<.001		N/A		
	51-64	0.3573		0.2173		1.644		0.10		N/A		
	≥65	0.9421		0.3045		3.094		0.002		N/A		

^a^Pr(>|z|): *P* value associated with the value in the z score column.

^b^N/A: not applicable.

#### Wait Times and Visit Duration

**Figure 1:**
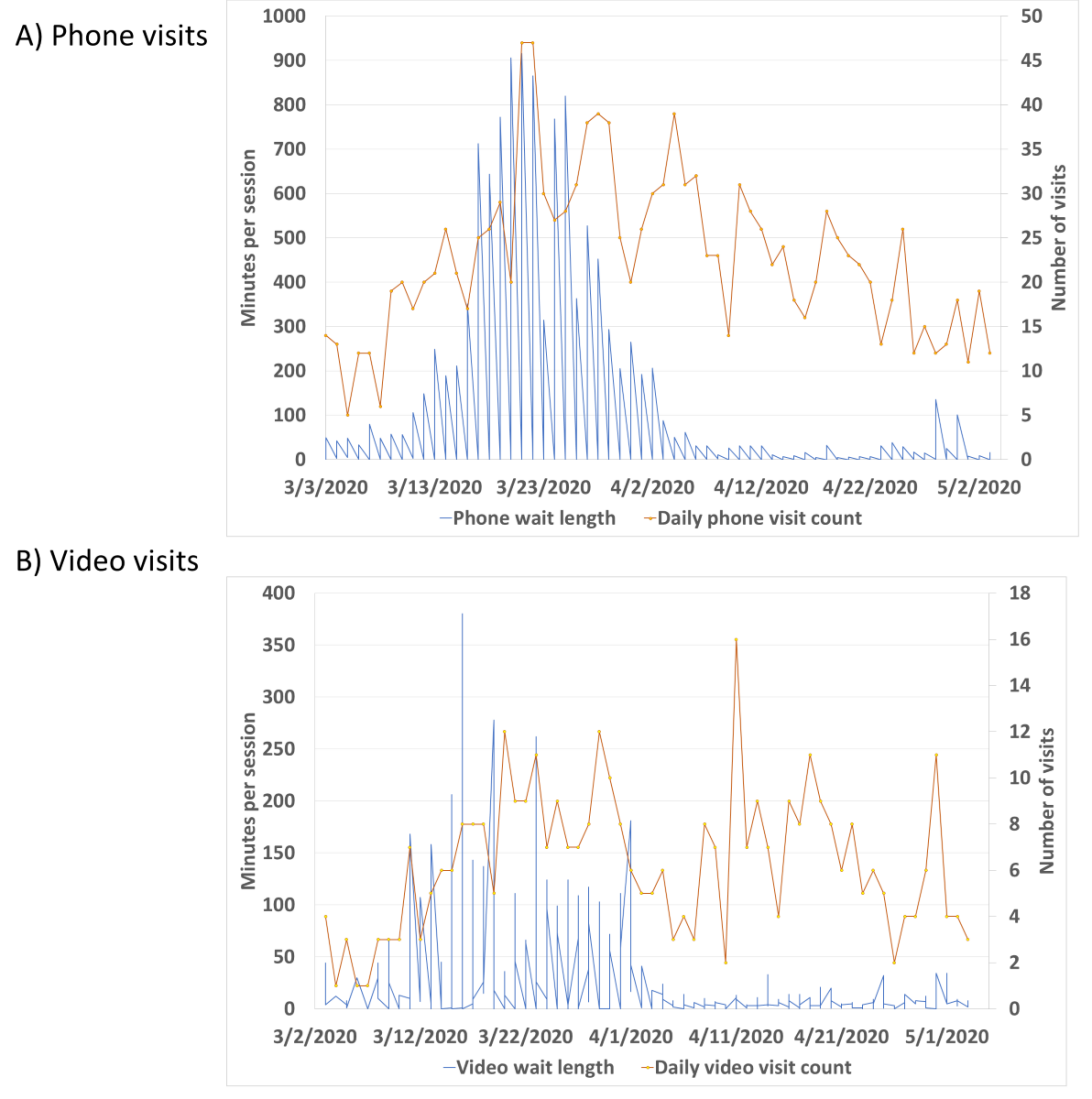
Comparison of (A) phone visit and (B) video visit wait times with a count of daily visits and physicians working.

The average wait time for patients to start their phone visits was 64.1 (SD 129.9) minutes, while the average wait time for patients with video visits was 24.6 (SD 45.6) minutes. The average visit duration for phone visits was 7.3 (SD 4.4) minutes, while the average visit duration for patients in video visits was 9.0 (SD 5.9) minutes. Significant differences existed between the average wait times and durations for phone and video visits (Welch *t* test *P*<.001 for both wait times and duration). For phone and video visits in this data set, the daily wait times for patients to see a physician across each modality are indicated in Figures 1A and 1B, respectively. The number of physicians working daily shown in these figures peaked at a maximum of 33 physicians on March 21 and 22. The number of phone sessions facilitated was also at its peak on these days at 47 phone visits. Phone users experienced the longest wait times in the second half of March, but both phone and video users experienced extended wait times in this same period compared to April.

### Telemedicine Utilization in Rural and Urban Areas

Of the 1080 NC zip codes, 262 (24.3%) had a low percentage of households with internet access, 277 (25.6%) had a medium percentage of households with internet access, and 269 (24.9%) had a high percentage of households with internet access. There were 272 (25.3%) zip codes with no internet access.

**Figure 2:**
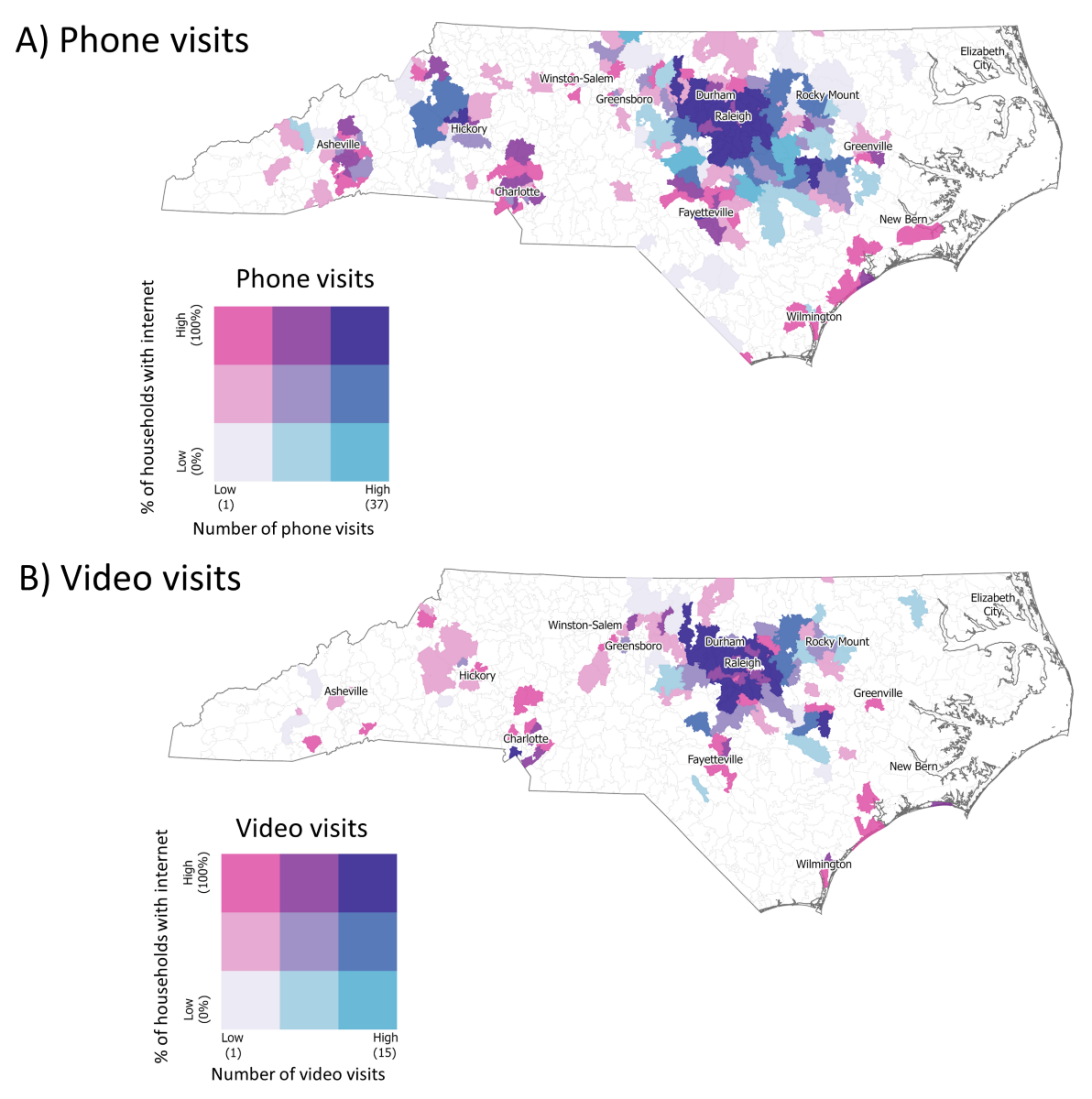
Comparison of (A) phone and (B) video telemedicine visits and the percentage of households in North Carolina with internet access based on American Community Service data.

The overall utilization of video visits was higher in areas with high percentages of households having internet access (Figure 2). Among the individuals from zip codes with low internet access there were 127 (83.5%) phone visits and 25 (16.5%) video visits. Zip codes with medium internet access had 367 (80.8%) phone visits and 87 (19.2%) video visits, and those with high internet access were 879 (76.2%) phone visits and 274 (23.8%) video visits.

Visits to the telemedicine-based clinic came from 431 (40%) unique NC zip codes. Of these, 251 (58.2%) were rural zip codes and 180 (41.8%) were urban zip codes (Figure 2). The density of the visits, shown in larger icons in Figure 2, originated mostly from major metropolitan areas like the state capitol or the Research Triangle Park. Phone visits provided further reach into areas with low internet access, while video visits mainly occurred in urban settings with high access to internet services.

Phone visits provided significantly more access to rural areas than video visits (*P*<.001). There were 1363 phone visits from patients in NC, with 780 (56.8%) being from rural areas and 583 (42.5%) from urban settings. There were 383 video visits from patients in NC, with 190 (49.2%) being from rural areas and 193 (50%) from urban settings.

Phone visits originated from 290 (26.9%) unique NC zip codes, of which 170 (58.6%) were from rural areas, 80 (27.6%) were from urban areas, and 40 (13.8%) were from out of state. Video visits occurred in 141 (32.4%) unique NC zip codes, of which 80 (56.7%) were from rural areas, 56 (39.7%) were from urban areas, and 5 (3.6%) were from out of state. Phone visits provided better reach into rural areas; however, video visits had widespread coverage, demonstrating the potential to complement phone visits in rural areas. Both phone and video visits within urban areas provided comparable coverage as expected.

## Discussion

### Principal Findings

We conducted a cross-sectional study of telemedicine urgent care visits completed through phone or video using a statewide, on-demand urgent care telemedicine clinic, focusing on demographics, utilization, and service characteristics. We observed significant differences in service characteristics between phone and video visits. The rate of medication prescription was much higher among phone visits compared to video visits. Patients had a higher probability of receiving a prescription during a phone visit, while the probability of receiving a prescription was lower during a video visit. Differences in gender, age, and telemedicine modality were associated with significant variations in prescription administration.

Similarly, significant differences in wait time and visit duration were observed between phone and video visits, where phone visits had higher wait times and longer visit durations. The high volume of requests for phone visits can justify the long wait. It was unclear if providers compensated for the long wait times by providing more visit time or if patients who waited longer had more questions based on the differences in visit durations.

Utilization of phone and video visits differed significantly. Women, insured patients, and those residing in rural areas preferred phone visits, while men, uninsured patients, and those residing in urban areas preferred video visits. Patients older than 65 years were equally split. The increase in video visits was due to pandemic-related cancellations of in-person appointments. Video visits were more common for children due to the need for clinical examination. Phone visits were more common in rural areas with no internet access for video visits. Rural patients preferred phone visits while urban patients preferred video visits. The reason for this preference is unclear. We suspect that a combination of privacy concerns, lack of confidence in their internet connection, and a lack of awareness may drive patients’ decisions; however, more investigation is needed [27,28].

Tying our findings to similar studies in the literature was a challenge because of a gap in studying the differences between telephone and video visits on the same outcomes [29]. Comparative studies have indicated that there has not been a meaningful difference between these modalities, having similar consultation session lengths, content, and perceived quality [30-32]. One study reported that older, rural, and ethnic minority patients were associated with lower utilization rates of video visits compared to phone visits [23]. A previous study reported that patients who had telephone visits had longer visit durations than those who had video visits [32], which contradicts our finding where video visits were longer in duration. A few studies have indicated increased utilization of telemedicine to trend toward women, with women being more likely to attend telephone-based interventions and to benefit from such interventions in the context of addiction treatment [33,34]. Moreover, another study showed that no major differences in utilization were found between video and telephone visits [31], which contradicts our findings demonstrating higher utilization of telephone visits compared to video visits.

Other studies explored telemedicine modalities separately demonstrating limitations due to selection bias in patient populations, such as including patients from a single hospital or clinic setting [13,14,16]. There is also concern that these studies often cater to specialized medical concerns or treatment options, which limits the demographic diversity of the patients recruited regarding factors such as age or gender, making the findings less generalizable [17,30]. Little was known regarding the patient characteristics of telephone or video telemedicine modalities across the rural-urban divide, patient insurance statuses, and prescriptions provided to the patients.

The COVID-19 policy waivers by the Center of Medicaid and Medicare and private insurers to include phone and video visits appear to be an effective decision that increased access and reduced disparities [35,36]. Additionally, this study shows that internet access is still limited in rural areas, which may limit the ability to conduct patient video visits, resulting in more phone visits. We recommend policymakers to continue to support video and phone visits equally, and we highlight the importance of building internet capacity within rural and vulnerable communities to expand the effective use of telemedicine.

### Limitations

This study had several limitations. We conducted a cross-sectional study as we could not randomize patients to a telemedicine modality due to the complexity of the process and given the sensitivity of COVID-19. In addition, the study was conducted over 2 months (March 3, 2020, to May 3, 2020) at the height of the pandemic with a limited amount of data; however, this reflected the initial response to the pandemic when telemedicine was the primary option for care. A large proportion of patients in this study were uninsured. Uninsured patients preferred telehealth during the initial phase of the pandemic due to the suspension of in-person visits and the shutdown of health care systems and primary care clinics, which are more expensive for uninsured patients compared to emergency departments [37]. This study did not include a comparison to in-person consultations because the health care system suspended all nonessential visits during the observed study period, starting on March 20, 2020. There were no data collected on race, ethnicity, or type of insurance used or covered, which could have added value to the findings of this study. The diagnosis type may confound the difference in prescription administration of phone and video visits. We could not merge the telemedicine data with the electronic health record data to assess the difference in documentation quality between phone and video visits. No information was available to determine if the visit wait times in the data set included those seeking a telemedicine visit immediately as opposed to at a later date. Wait times could be separated for those seeking immediate appointments to improve our findings. Physician-level data was not accessible, limiting our assessment of factors such as clinician preparedness. Finally, the study findings were limited to 1 site, and so the generalizability to other settings is limited.

### Conclusion

Our study analyzed the use of phone and video visits at a telemedicine clinic during the COVID-19 pandemic. We discovered that providing patients with a variety of phone and video options was beneficial for many patient groups, especially those in rural or low-bandwidth areas. Gender, age, and insurance status were also factors affecting usage. Moreover, we observed differences in prescription administration between the 2 modalities that require further investigation. Our findings indicate that phone visits were more prevalent in rural regions compared to urban areas. To promote telemedicine adoption and quality, we must work toward improving internet infrastructure in rural areas, educating patients on selecting the appropriate modality, and establishing equitable service policies for phone and video visits.

## Abbreviations

ACS: American Community Service
NC: North Carolina
VUC: Virtual Urgent Clinic
ZCTA: Zip Code Tabulation Area
